# Exploring the Intersection of Autism, Theory of Mind, and Driving Performance in Novice Drivers

**DOI:** 10.1007/s10803-024-06526-9

**Published:** 2024-08-28

**Authors:** Abigale Plunk, Amy S. Weitlauf, Zachary Warren, Daniel Levin, Nilanjan Sarkar

**Affiliations:** 1 Department of Electrical and Computer Engineering, Vanderbilt University, Olin Hall 101, 2400 Highland Ave, Nashville, TN 37212 USA; 2 Department of Pediatrics, Vanderbilt University Medical Center, Nashville, TN USA; 3 Department of Psychiatry and Behavioral Sciences, Vanderbilt University Medical Center, Nashville, TN USA; 4 Department of Special Education, Vanderbilt University, Nashville, TN USA; 5 Department of Psychology and Human Development, Vanderbilt University, Nashville, TN USA; 6 Department of Mechanical Engineering, Vanderbilt University, Nashville, TN USA

**Keywords:** Autism spectrum disorder, Theory of mind, Driving, Decision-making

## Abstract

This study explores the intersection of Theory of Mind (ToM) abilities and driving performance among novice drivers, with a focus on autistic individuals. The purpose is to investigate how ToM deficits may impact driving behaviors and decision-making, ultimately informing the development of tailored interventions and training programs for autistic drivers. We conducted a series of driving simulations using a custom-built driving simulator, capturing multimodal data including driving performance metrics, attention allocation, and physiological responses. Participants were categorized based on NEPSY scores, which assess ToM abilities, and self-reported autism spectrum disorder (ASD) diagnosis. Driving tasks were designed to simulate real-world scenarios, particularly focusing on intersections and merging, where ToM skills are crucial for safe navigation. Our analysis revealed differences in driving behaviors among participants with varying ToM abilities as determined through the NEPSY. Participants with lower NEPSY scores exhibited less smooth driving behaviors, increased risk-taking tendencies, and differences in attention allocation compared to those with higher scores. Alternatively, individuals with ASD displayed comparable driving patterns overall. ToM abilities influence driving behaviors and decision-making, particularly in complex social driving scenarios. Tailored interventions addressing ToM deficits and stress management could improve driving safety and accessibility for autistic individuals. This study underscores the importance of considering social cognitive factors in driving education and licensure pathways, aiming for greater inclusivity and accessibility in transportation systems.

**Supplementary Information:**

The online version contains supplementary material available at 10.1007/s10803-024-06526-9.

Obtaining a driver’s license offers independence, allowing people to overcome transportation limitations and take on new responsibilities. However, this milestone is less attainable for many autistic teenagers and young adults. Although most autistic^1^ individuals state their intention to drive (Curry et al., 2018), only 24% of autistic adults are licensed to drive compared to 75% of the population as a whole (Lindsay, 2017). This disparity not only affects autonomy but also poses significant challenges in accessing employment opportunities, affecting financial independence.

Multiple factors contribute to the lower driver’s license acquisition rate among autistic individuals. Autism is characterized by executive functioning deficits, including challenges in attention and processing speed, which are critical skills for safe driving (Classen et al., 2013; Kristina Patrick et al., 2018). Additionally, driving is an inherently social task that entails understanding the intentions and behaviors of other drivers, which relies heavily on theory of mind (ToM)– the ability to attribute mental states to oneself and others (Baron-Cohen et al., 1985; Bishop et al., 2017). ToM deficits, commonly observed in autistic individuals, may hinder the ability to accurately interpret and anticipate the actions of the other drivers on the road amplifying the risks associated with driving. Furthermore, hyper or hypo-sensitivities, difficulties with appropriately directed eye gaze, and the high prevalence of co-morbidities such as anxiety can make the prospect of driving intimidating and learning to drive challenging (Baron-Cohen et al., 2009; Daly et al., 2014). These multifaceted barriers highlight the necessity for tailored driving training approaches for autistic teens and young adults.

Conventional driver education, often conducted through on-road training, may not adequately address the unique challenges faced by autistic individuals (Almberg et al., 2017). On-road training lacks the controlled environment necessary for safe practice and comprehensive data collection (Randall et al., 2021). Without the ability to simulate diverse driving scenarios while recording performance, attentional, and physiological data, traditional training methods may overlook critical aspects of skill development and fail to address the specific needs of autistic learners. In contrast, driving simulators offer a safe, controlled setting where young drivers can develop essential driving skills while building confidence and mitigating the risks associated with on-road driving (Campbell et al., 2016; Ekeh et al., 2013; Eriksson et al., 2018; Hirsch & Bellavance, 2017; Martín-delosReyes et al., 2019; Rosenbloom & Eldror, 2014).

In the past decade, there has been a notable increase in research utilizing driving simulators to investigate the driving attributes of autistic individuals. Cox et al. found that novice ASD drivers demonstrated poorer overall driving ability relative to novice drivers without ASD [14]. Similarly, Patrick et al. found that young adults with ASD may encounter greater challenges with fundamental driving skills compared to their peers, especially during the initial phases of driver training (Kristina Patrick et al., 2018). These difficulties tend to escalate as driving tasks become more intricate. However, Ross et al. noted that novice ASD drivers in their study displayed driving proficiency and depending on the performance metric ranked comparably to the non-ASD drivers (Veerle Ross et al., 2019). Additionally, Chee et al. found autistic drivers appeared to be safer with respect to following distance but did show poorer performance in response to complex traffic situations (Chee et al., 2019). Nonetheless, these studies primarily focused on driving performance metrics, failing to capture indicators of stress or attentional patterns, which are equally vital for safe driving (Matthews et al., 1991, 1996; Pavlidis et al., 2016). Reimer et al. expanded on driving performance metrics to include physiology and eye tracking (Reimer et al., 2013). Their findings revealed nominally higher and unvaried heart rates among ASD drivers compared to non-autistic drivers, alongside divergent gaze patterns, suggesting decreased attention to the roadway. Similarly, Cox et al. found greater anxiousness in autistic drivers when incorporating heart rate and galvanic skin response (Cox et al., 2020).

Despite being a growing area of research, there remains a significant gap in understanding driving differences among autistic individuals to develop a more personalized training program tailored to the needs of autistic drivers. Furthermore, previous studies overlook the importance of ToM, a crucial aspect of social cognition essential for safe driving.

The ability to drive extends beyond the mechanical operation of the vehicle; it requires cognitive processes and social interactions. Cognitive processes crucial for safe driving include attention, perception, memory, decision-making, problem-solving, planning, and anticipation (Tapia & Duñabeitia, 2023). Additionally, driving is a social task, requiring drivers to interact with other road users cooperatively and communicatively (Swan & Owens, 1988; Wilson et al., 2018). Successful driving relies on effective unspoken social understanding of other road users’ intentions. Drivers must allocate their attention effectively to monitor the road, interpret visual and auditory cues, recall traffic rules, make split-second decisions, solve problems, and anticipate potential hazards (Trick * et al., 2004). The intricate combination of cognitive functions and social interactions highlights the importance of ToM. ToM may enable drivers to predict the intentions and behaviors of fellow road users, facilitating smooth and safe interactions on the road.

Given the potential importance of ToM in driving, it is essential to develop driving tasks that specifically assess individuals’ ability to attribute mental states to themselves and others. Two examples of driving tasks that require ToM skills include navigating intersections and merging. When approaching an intersection, drivers must pay attention to other road users, such as oncoming traffic and pedestrians, and make decisions based on their behaviors. Similarly, when merging, drivers must gauge the speed, trajectory, and intention of neighboring vehicles and adjust their driving behavior accordingly. Successfully navigating these situations requires the ability to interpret subtle cues to predict the actions of the drivers around you rather than making assumptions based upon what you yourself would do.

In this paper, we utilize multimodal data encapsulating driver performance, attention, and stress collected in a custom-driving simulator. We build on prior research by developing a series of merging and intersection driving tasks that rely on ToM. These tasks are designed to simulate real-world driving scenarios, providing a comprehensive understanding of how ToM deficits may impact driving performance in autistic individuals. Our study seeks to provide empirical insights into driving challenges among autistic individuals. In doing so, we aim to inform the development of targeted interventions and training programs to improve driving safety and independence within this population.

## Methods

### Experimental Setup

We utilized a custom driving simulator named SIAD, which stands for Simulator for Individualized and Adaptive Driving. SIAD consists of a Logitech G920 steering wheel and pedals, a Thrustmaster TH8A gear shifter, a Tobii Pro X3-120 eye tracker, a web camera, an EmotiBit wearable biometric sensor, and custom driving software developed in Unity3D. The experimental setup is shown in Online Resource 1. Online Resource 1 does not include the EmotiBit sensor, however, participants wore the sensor on their non-dominant arm. Previous work developed this driving simulator and investigated affect recognition, visual attention, and cognitive state measurement in autistic drivers (Bian et al., 2013, 2019; Wade et al., 2016, 2017; Zhang et al., 2017).

Using a custom driving simulator like SIAD offers advantages over off-the-shelf options. While some off-the-shelf driving simulators allow for variation in task design, this custom simulator allows for full control over the simulation environment. SIAD can be tailored to the specific needs of each study allowing us to implement the intersection and merging tasks described in the following section. Additionally, we can integrate advanced data collection such as physiology and eye tracking directly into the system rather than recording it concurrently. This customization is vital for accurate and consistent data collection on driving behavior.

### Driving Task Design

Understanding the relationship between Theory of Mind (ToM) and driving performance is crucial for enhancing road safety, especially among young drivers with and without autism. To investigate this relationship comprehensively, we developed a series of intersection and merging driving tasks that rely on ToM within our custom driving simulator, SIAD (Simulator for Individualized and Adaptive Driving), built using the Unity3D engine. These tasks were designed to simulate real-world driving scenarios and assess participants’ decision-making processes in various social and environmental contexts. Leveraging the flexibility of Unity3D, we were able to realize the designs by developing roadways and integrating dynamic elements such as pedestrians and other vehicles into the simulation.

**Fig. 1:**
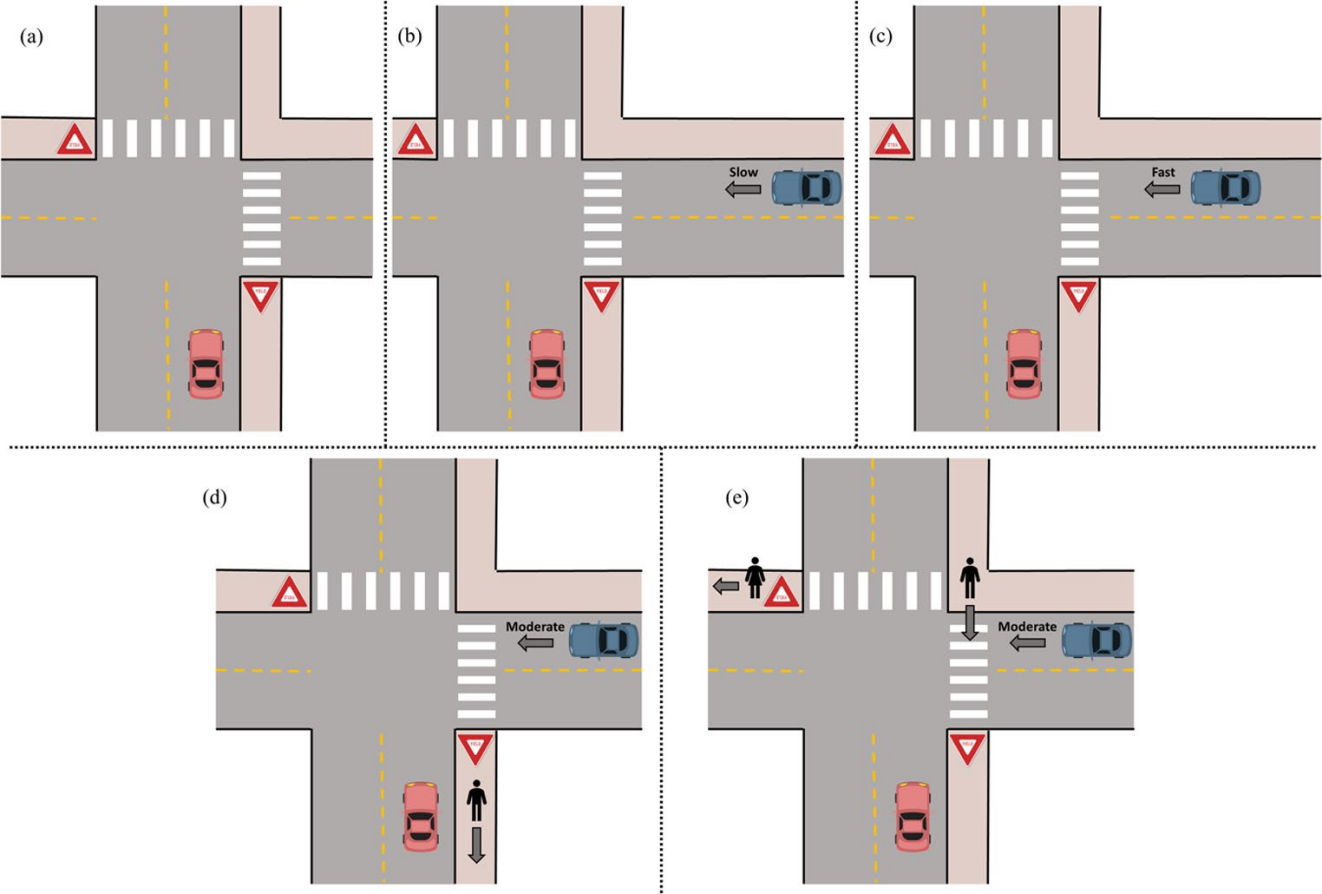
Illustration of intersection driving tasks: (**a**) demonstrates the first intersection task with no other vehicles or pedestrians, (**b**) adds a slow-moving oncoming vehicle, (**c**) increases the speed of the oncoming vehicle, (**d**) adds a pedestrian that does not affect the task, and (**e**) increases the number pedestrians and has one crossing in the path of the oncoming vehicle

Table 1; Fig. 1 demonstrate the intersection driving tasks. As shown in row 1 of the table, in the first intersection task, the driver approaches a yield sign, and there are no other cars or pedestrians on the road. Following this, in the second task, there is now an oncoming car represented in blue. This task is split into two variants, as detailed in rows 2a and 2b - in the first variant the blue car is going slow and far from the intersection, while in the second variant, the blue car is traveling faster and closer to the intersection. The two variants of the second task require the participant to infer the speed and distance of the blue car to inform their action. In the third task, shown in row 3, we increase the complexity by adding a pedestrian, though the pedestrian does not directly affect the task. In the final intersection task, detailed in row 4, there are now multiple pedestrians, and one of the pedestrians blocks the path of the blue car, causing it to come to a stop and wait for the pedestrians to safely cross.

**Table 1:** Intersection driving tasks

Task	Description
1 (Fig. 1a)	The driver (red car) will approach a yield sign, and there will be no other cars or pedestrians on the road.
2a (Fig. 1b)	There will be an oncoming car represented in blue, going slow and far from the intersection. It would be safe for the participant to go in front of it after yielding.
2b (Fig. 1c)	The blue car will be traveling faster and will be closer to the intersection. If the participant either does not yield or proceeds in front of the blue car after yielding, they will likely get hit.
3a (Fig. 1d)	The complexity is increased by adding a pedestrian. However, the pedestrian does not directly affect the task. The blue car is driving moderately and is a moderate distance away from the intersection. The participant would have time to yield and go before the blue car.
3b (Fig. 1e)	There are now multiple pedestrians, and one of the pedestrians blocks the path of the blue car. The blue car will come to a stop and wait for the pedestrians to safely cross the road before resuming motion.

**Fig. 2:**
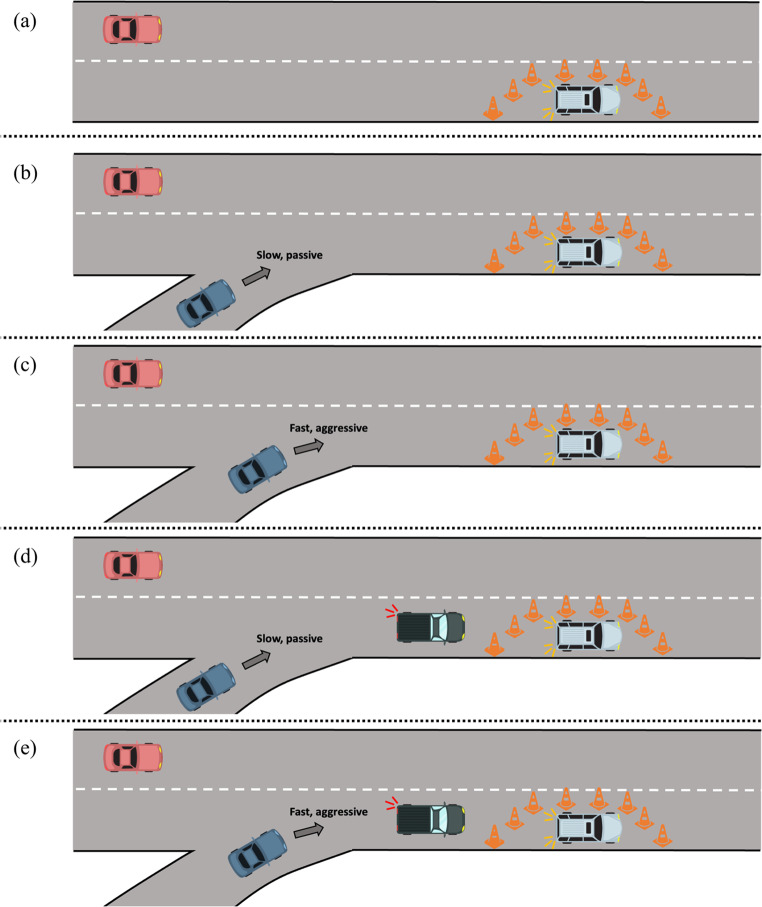
Illustration of the merging driving tasks: (**a**) demonstrates the first merging task with only an obstacle, (**b**) adds a slow-moving passive oncoming vehicle, (**c**) increases the speed and changes the driving style of the oncoming vehicle, (**d**) repeats task b and adds a truck, and (**e**) repeats task c and adds a truck

Table 2; Fig. 2 demonstrate the merging driving tasks. As in the intersection tasks, the red vehicle is driven by the participant. In the merging tasks, the participant begins the task in the second to right lane and is asked to remain in that lane for the entirety of the task. They will come across two broken-down vehicles in the right lane surrounded by cones. The first obstacle is always a control. In the first task, the second obstacle is also a control. The second and third tasks have a car, demonstrated in blue, merging onto the highway immediately before the obstacle. The speed and driving style of this car varies between tasks. In the second one, the blue car is driving slowly in a passive style. Whereas, in the third task, the blue car is driving quickly and aggressively. The final two tasks add a second vehicle, demonstrated as a green truck, that does not directly affect the task. Again, the blue car drives slowly and passively in task four, while the blue car drives quickly and aggressively in task five.

**Table 2:** Merging driving tasks

Task	Description
1 (Fig. 2a)	The driver (red car) will approach an obstacle in the other lane on the side of the road, and there will be no other cars on the road.
2a (Fig. 2b)	An oncoming car will be represented in blue, merging onto the highway immediately before the obstacle. The blue car is driving slowly in a passive style.
2b (Fig. 2c)	An oncoming car will be represented in blue, merging onto the highway immediately before the obstacle. The blue car is driving quickly and aggressively.
3a (Fig. 2d)	Repeats task 2a and adds a green truck that does not directly affect the task.
3b (Fig. 2e)	Repeats task 2b and adds a green truck that does not directly affect the task.

In addition to the design considerations for the intersection and merging driving tasks, our study employed a randomized task order approach to enhance robustness. With a total of 10 intersection tasks split into two sets of five intersection tasks and five merging tasks, we organized them into three distinct sets, each comprising five tasks. Within each set, the order of tasks remains constant, following the sequence shown in Tables 1 and 2 that increases in complexity. However, to mitigate potential order effects and control for any biases introduced by task sequencing, we adopted a randomized approach at the set level. By randomizing the order of these three sets, we minimized the influence of task order on performance outcomes. Through this methodological approach, we aimed to capture the nuanced interactions between cognitive processes, social understanding, and driving performance, thereby advancing our understanding of the complex dynamics involved in safe driving.

### Participant Information

We recruited 25 participants for this study, including 13 who were self-reported as having ASD and 12 without ASD. We were looking for teenagers and young adults between the ages of 13 and 22 who were about to start learning to drive, learning to drive, or who were recently licensed. Participant demographics are shown in Table 3. All participants provided consent and assent in accordance with Institutional Review Board guidelines. Participants received a gift card as compensation for their time. All participants but one completed all driving tasks. However, we utilized the data collected from their partial involvement in the study.

**Table 3:** Participant demographics

	ASD	No-ASD	*p*-value
**# of Participants**	15	10	
**Gender**			
Male	9	5	
Female	5	5	
Non-binary	1	0	
**Age**			
Mean ± STD	16.3 ± 2.1	15.0 ± 1.6	0.0996
**Social Communication Questionnaire (SCQ)**			
Mean ± STD	16.6 ± 10.2	6.5 ± 7.6	**0.0140**
**Social Responsiveness Scale (SRS)**			
Mean ± STD	81.7 ± 39.6	33.3 ± 29.1	**0.0031**
**SRS T-Score**			
Mean ± STD	65.1 ± 14.5	46.7 ± 11.2	**0.0024**
**SRS Social Awareness**			
Mean ± STD	57.0 ± 13.1	42.7 ± 9.43	**0.0070**
**NEPSY ToM**			
Mean ± STD	18.1 ± 4.7	18.6 ± 4.5	0.7778
**Has Permit/License**			
Yes (%)	33%	30%	

*Significant Values (*p* < 0.05) in bold

#### Autism Characteristics

The Social Responsiveness Scale, second edition (SRS-2) (Constantino & Gruber, 2012) and the Social Communication Questionnaire, Lifetime Version (SCQ) (Berument et al., 1999; Rutter et al., 2003) were completed for each participant by his/her parent to quantify ASD symptoms. Our analysis using correlation coefficients found a significant correlation between self-reported autism diagnosis and scores obtained from both the Social Communication Questionnaire (SCQ) and the Social Responsiveness Scale (SRS). The correlation coefficient was 0.49 (*p* = 0.014) for the SCQ and 0.58 (*p* = 0.0024) for the SRS T-Score, indicating a moderate to strong positive correlation between self-report and standardized assessment tools for autism diagnosis.

#### Theory of Mind

Participants were administered the Theory of Mind subtest of the NEPSY-II (Korkman et al., 2007) under the guidance of a licensed psychologist. The NEPSY-II (A Developmental Neuropsychological Assessment) is a widely used test battery designed to assess neuropsychological development in children and adolescents. The Theory of Mind (ToM) subsection within the NEPSY evaluates the individual’s ability to understand the thoughts, beliefs, intentions, and emotions of oneself and others.

The scoring on the ToM subsection of the NEPSY typically involves assessing the individual’s performance on various tasks related to theory of mind, such as understanding false beliefs, recognizing emotions in others, and comprehending social cues. Because some of our participants’ age exceeded the validated age range for the NEPSY-II (3–17 years old), we used raw scores to compare ToM performance across participant groups. Raw scores on this subtest range from 0 to 22, with 22 being a perfect score.

#### Driving Theory of Mind Questionnaire

We designed a questionnaire to explore Theory of Mind (ToM) in driving situations. It asks questions about participants’ thoughts and feelings regarding other cars and pedestrians on the road. The questionnaire utilized a Likert scale ranging from − 10 to 10, where respondents indicated their level of agreement from ‘none’ to ‘a lot’ to gauge how much individuals consider, predict, and are impacted by the actions of others while driving. This questionnaire offers a way to understand our participant’s ToM in the context of driving behavior. This questionnaire is shown in Online Resource 2.

### Procedures

All participants attended a single session lasting approximately 90 min. Following consenting procedures, the EmotiBit physiology wristband was put on the participant before they completed the NEPSY ToM instrument. Following completion, the driving simulator playseat was adjusted to the participant based on their height. Then, the Tobii eye-tracking device was calibrated to the participants’ eyes using a nine-point calibration procedure, and we began recording physiology data.

After calibration, the participants completed two driving tutorials. This was important as many of the participants had never driven before. The first was an acceleration and braking tutorial which took around 2 min to complete. It required the participant to reach and maintain three different speed intervals (30 mph, 45 mph, and 60 mph) before slowing down to a complete stop. The second tutorial was a turning tutorial. This tutorial guided drivers through the city and required them to make 3 right turns and 3 left turns after stopping at stop signs and looking both ways which can be done with the directional buttons on the steering wheel. Following the two driving tutorials, participants continued habituating to the simulator and practicing in a guided drive around the city and on the highway. This guided drive allowed them to practice slowing down at yield signs, stopping at stop signs, and merging onto the highway.

Following this practice period, which lasted approximately ten minutes, participants proceeded to the 15 ToM driving tasks described previously. Upon completion of the 15 tasks, the driving ToM questionnaire we developed was administered.

### Data Collection

**Fig. 3:**
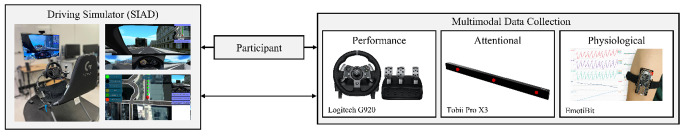
Driving simulator data collection framework

We collected quantitative data from multiple modalities to capture performance, attentional, and physiological information. Performance data was collected through the Logitech steering wheels and pedals. This modality can indicate sudden braking, irregular acceleration, and erratic steering. Attentional data was captured through a Tobii Pro X3 which has a sampling rate of 120 Hz. Through the Unity API, we got the x and y-screen coordinates of the participant’s gaze. Physiological data was captured using an EmotiBit wearable sensor. The EmotiBit measures electrodermal activity (EDA), multi-wavelength photoplethysmography (PPG), skin temperature, and motion with a 9-axis IMU. From PPG, we derive heart rate variability (HRV). The driving simulator and data collection framework are shown in Fig. 3.

### Metrics

We analyzed the driving in the ToM tasks based on three primary quantitative domains from the driving study: performance, attention, and physiology. We additionally analyzed driving outcomes based on decisions participants made in the tasks and their responses to the driving ToM questionnaire.

#### Performance

Performance metrics are categorized into intersection results and merging results, with a focus on steering wheel rotation, acceleration/deceleration, speed, and number of wrecks.

Steering wheel rotation provides insights into the driver’s control over the vehicle. The ToM tasks were intentionally designed to require little to no turning to decrease the complexity allowing us to focus on the decisions the drivers made. Therefore, we should not observe significant steering wheel rotation. Monitoring acceleration and deceleration patterns offers valuable information about driving behavior including smooth driving style. In the following sections, we will jointly look at the percentage of the time in the tasks that participants were accelerating in addition to the number of “hard accelerations”. We defined a “hard acceleration” as an acceleration exceeding 0.5 g which was decided on based on literature (Klauer et al., 2009; Simons-Morton et al., 2009). Speed is a fundamental aspect of driving performance, directly impacting safety. By analyzing speed, researchers can assess whether drivers adhere to speed limits and adjust their driving behavior appropriately based on social stimuli. Finally, the occurrence of wrecks serves as a critical safety indicator, reflecting the driver’s ability to anticipate the actions of other drivers on the road.

#### Attention

Attention is quantified using the x- and y-coordinates of gaze supplied by the Tobii Pro X3 eye tracker. We analyze the percentage of time participants focus on the road and on the speedometer to gauge driver attention.

#### Physiology

From the EmotiBit Physiological sensor, we collect photoplethysmography (PPG) data. One participant’s data was omitted from this analysis due to noise from the sensor. We use Kubios HRV software for heart rate variability (HRV) analysis based on the PPG data collected (Lipponen & Tarvainen, 2019; Niskanen et al., 2004; Tarvainen et al., 2014). We utilize HRV to analyze the functioning of the autonomic nervous system in our experiment. Kubios calculates indices of parasympathetic nervous system (PNS) functioning, sympathetic nervous system (SNS) functioning, and stress. The PNS is responsible for “rest and digest” while the SNS is responsible for “fight or flight” meaning that the SNS index will show us how the participants *respond* to the stimuli and the PNS index will show us how the participants *recover* from the stimuli (Migovich et al., 2022).

## Results

In this section, we will present results based on the metrics defined above. We found no significant differences in NEPSY scores based on self-reported autism diagnosis. Therefore, we additionally grouped participants by their NEPSY score to investigate the impact of ToM on driving.

We split the participants into 3 groups based on ToM. “High NEPSY” indicates their score was above the median which was a score above 20. “Medium NEPSY” indicates their score was between the median and the lower quartile which was a score between 15 and 20. Finally, “Low NEPSY” indicates their score was below the lower quartile which was a score below 15. The groups are not equally distributed. 12 participants are included in the “High NEPSY” group, 7 participants in the “Medium NEPSY” group, and 6 participants in the “Low NEPSY” group. The participant who discontinued before the conclusion of the study was part of the “Low NEPSY” group.

### Performance

In this section, we present performance metrics categorized into intersection results and merging results.

#### Intersections

**Fig. 4:**
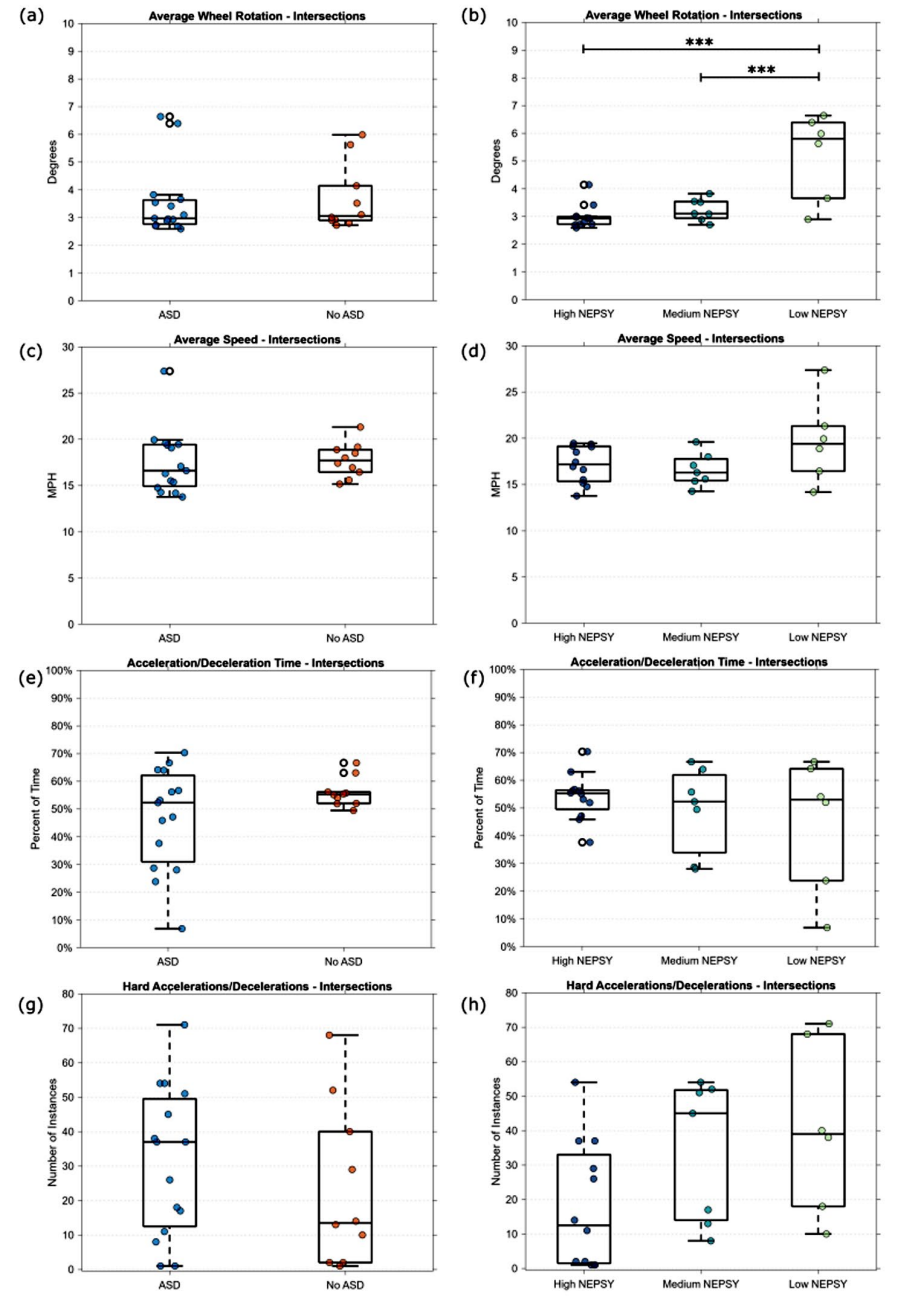
Performance metrics for the Intersection tasks based on self-report of ASD and NEPSY score grouping. (***p < 0.001)

Figure 4 shows the average wheel rotation, speed, and acceleration performance metrics of each participant across all the intersection tasks. The speed limit in these tasks was 25 MPH and no turning was required in the task. Each of the tasks included two intersections with yield signs so we would expect the average speed across the entire level to be less than the speed limit. As shown in Fig. 4a and c, there were minimal differences in wheel rotation and speed when the groups were split based on self-reported ASD. However, in Fig. 4b, a one-way ANOVA test showed a significant difference in wheel rotation in the “Low NEPSY” group (F(2, 22) = 15.33, *p* < 0.001). Groupwise comparisons with a Tukey correction showed that the wheel rotation for the “Low NEPSY” group was significantly higher than both the “Medium NEPSY” (*p* < 0.001) and the “High NEPSY” (*p* < 0.001), while no significant difference were found between the “Medium NEPSY” and “High NEPSY” groups. We observed a slightly increased median average speed across the participants in the “Low NEPSY” group in Fig. 4d. Figure 4e and f show the percentage of time the participants were accelerating or braking in the intersection tasks. Figure 4g and h, show the number of instances of accelerations or decelerations greater than 10 mph/s. We saw a much wider range of time spent accelerating and braking among the participants in the ASD group compared to the No ASD group. Additionally, we saw an increase in the number of hard accelerations/decelerations in the ASD group. The 3 groups spent a similar amount of time braking and accelerating, however, the “Medium NEPSY” and “Low NEPSY” groups had more instances of hard accelerations and decelerations than the “High NEPSY” group.

#### Merging

**Fig. 5:**
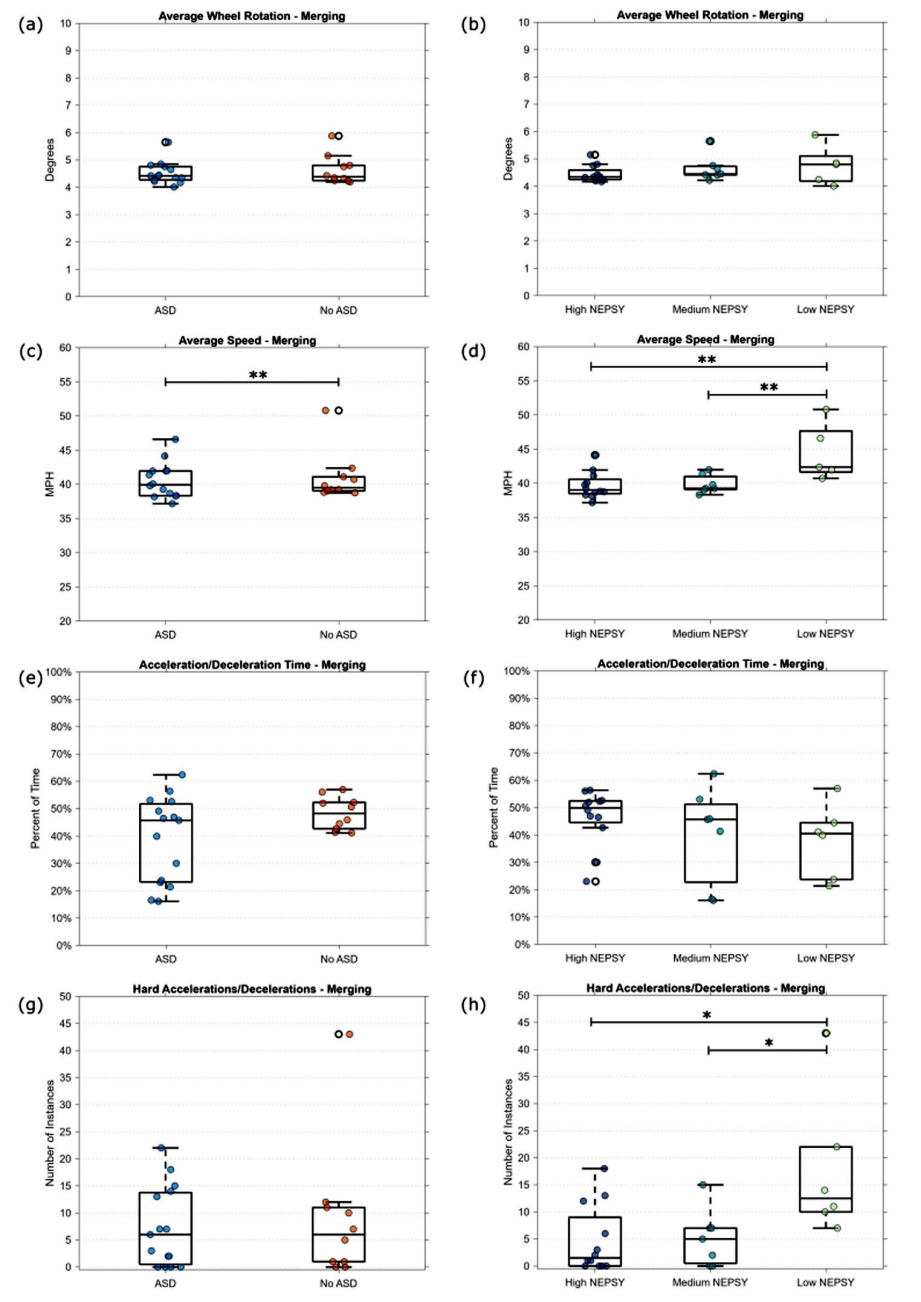
Performance metrics for the Merging tasks based on self-report of ASD and NEPSY score grouping. (**p < 0.01, *p < 0.05)

Figure 5 shows the average wheel rotation, speed, acceleration, and deceleration performance metrics of each participant across all the merging tasks. The speed limit in these tasks was 45 MPH and no turning was required in the task. Each of the tasks included an obstacle on the side of the road with a car showing the intent to merge into the participant’s lane to avoid the obstacle, so we would expect the average speed across the entire level to be less than the speed limit. As shown in Fig. 5a and c, there were minimal differences in wheel rotation and speed when the groups were split based on self-reported ASD. Similarly, in Fig. 5b, we observed minimal differences in wheel rotation when splitting the groups based on NEPSY score. However, we saw an increased average speed in the “Low NEPSY” group (F(2, 22) = 7.78, *p* < 0.01) in Fig. 5d. Groupwise comparisons with a Tukey correction showed that the average speed for the “Low NEPSY” group was significantly higher than both the “Medium NEPSY” (*p* < 0.01) and the “High NEPSY” (*p* < 0.01), while no significant difference was found between the “Medium NEPSY” and “High NEPSY” groups.

Figure 5e and f show the percentage of time the participants were accelerating or braking in the merging tasks. Figure 5g and h, show the number of instances of accelerations or decelerations greater than 10 mph/s. Similar to the intersection tasks, in Fig. 5e, we observed a much wider range of time spent accelerating and braking among the participants in the ASD group compared to the No ASD group. However, we observed a similar number of hard accelerations/decelerations in the ASD and No ASD groups in Fig. 5g. We observed a different trend when grouping based on NEPSY scores. There is a slight negative trend in the amount of time the 3 groups spend accelerating and braking in Fig. 5f, with the “Low NEPSY” group spending less time braking and accelerating than the other two groups. However, in Fig. 5h, we observed an increase in the number of hard acceleration and braking occurrences in the “Low NEPSY” group (F(2, 21) = 5.71, *p* < 0.05).

#### Wrecks

**Fig. 6:**
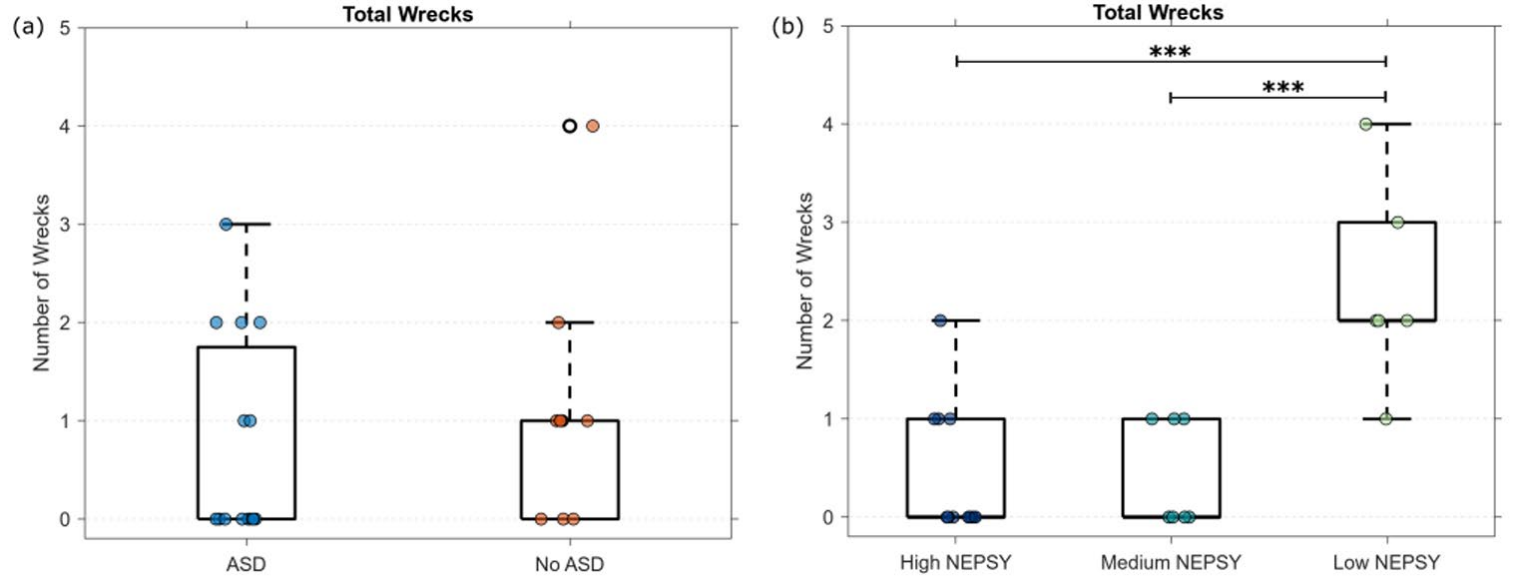
Total number of wrecks based on self-report of ASD and NEPSY score grouping. (***p < 0.001)

The final performance metric we analyze is the number of wrecks across all the tasks. In this metric, we only count one wreck per task because the majority of the time the task was no longer able to be completed after a wreck occurred. Additionally, we only count wrecks with other vehicles. Figure 6 shows the number of wrecks based on the groups. It is important to note that potential wrecks are not unexpected as this was many of the participant’s first experience driving in any capacity, and we are having them complete complex social driving scenarios. In Fig. 6a, we observed a similar number of wrecks between the ASD and No ASD groups. However, in Fig. 6b, we observed that the “Low NEPSY” group had more wrecks than the other two groups (F(2, 22) = 15.33, *p* < 0.001). Groupwise comparisons with a Tukey correction showed that the “Low NEPSY” group had significantly more wrecks than both the “Medium NEPSY” (*p* < 0.001) and the “High NEPSY” (*p* < 0.001), while no significant difference was found between the “Medium NEPSY” and “High NEPSY” groups.

### Attention

**Fig. 7:**
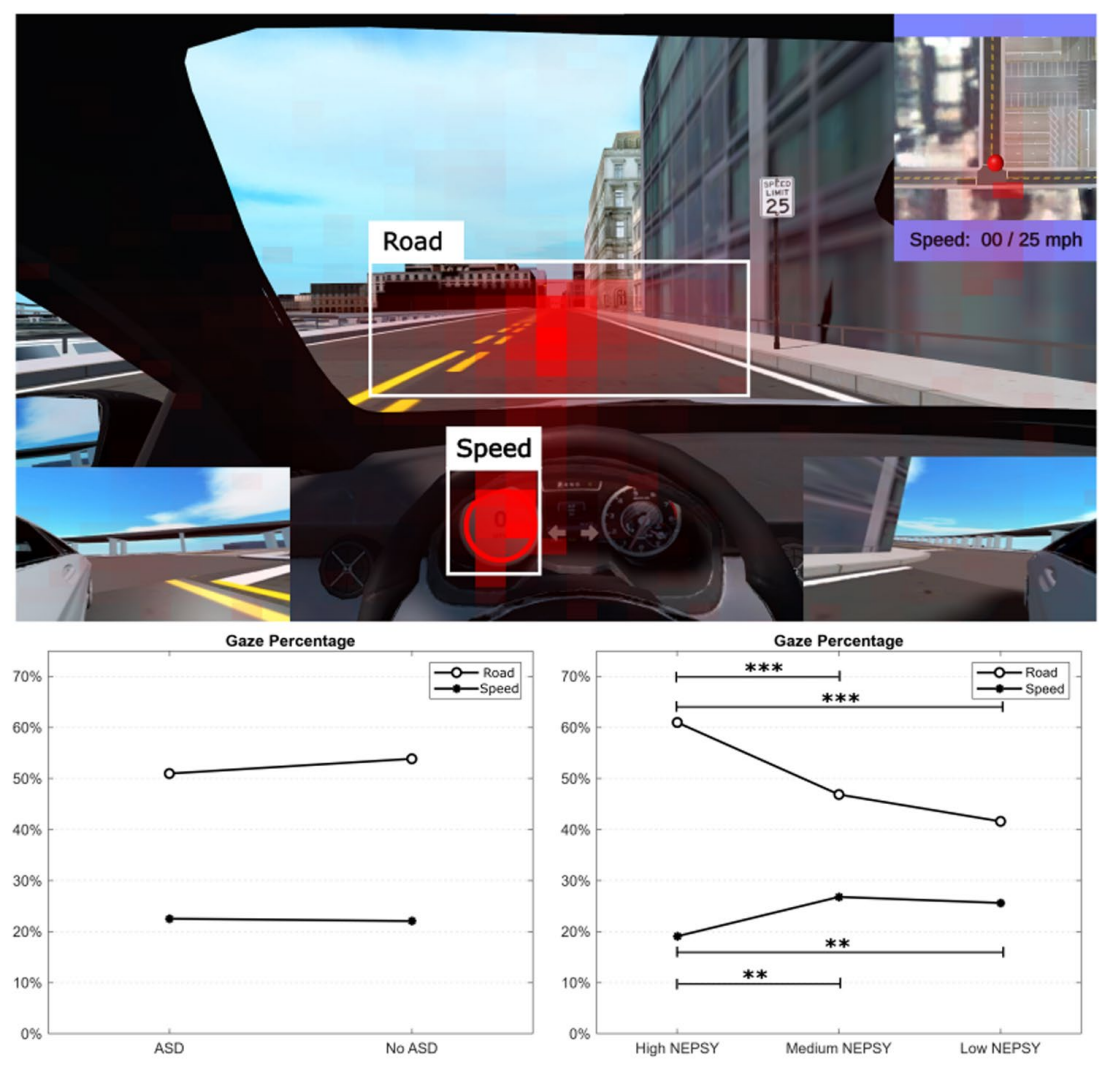
Attention heat map based on eye gaze with gaze percentages for Box 1, focus on the road, and Box 2, focus on the speedometer, across groups based on self-report of ASD and NEPSY score. (***p < 0.001)

In this section, we look at the attention of the participants across all of the tasks. We additionally completed the analysis for the intersection and merging tasks separately but found negligible differences. We plotted a heatmap of the x- and y-coordinates of the gaze collected using the Tobii Pro X3. We found that the vast majority of the attention was on the road, as expected, and the speedometer so we defined two regions of interest (ROI) shown in Fig. 7 as “Road” and “Speed”.

Next, we calculated the percentage of time each participant looked at these two ROIs across all the tasks. As shown in Fig. 7, there are negligible differences in the amount of time the group with ASD looked at the two ROIs compared to the No ASD group. However, we saw a decreased attention on the road in the “Low NEPSY” group (F(2, 42) = 20.86, *p* < 0.001) in Fig. 7. Groupwise comparisons with a Tukey correction showed that the average attention on the road for the “High NEPSY” group was significantly higher than both the “Medium NEPSY” (*p* < 0.001) and the “Low NEPSY” (*p* < 0.001), while no significant difference was found between the “Medium NEPSY” and “Low NEPSY” groups. We also see an increased attention on the speed in the “Low NEPSY” group (F(2, 42) = 8.08, *p* < 0.01) in Fig. 7. Groupwise comparisons with a Tukey correction showed that the average attention on the speedometer for the “High NEPSY” group was significantly lower than both the “Medium NEPSY” (*p* < 0.01) and the “Low NEPSY” (*p* < 0.01), while no significant difference was found between the “Medium NEPSY” and “Low NEPSY” groups.

### Physiology

**Fig. 8:**
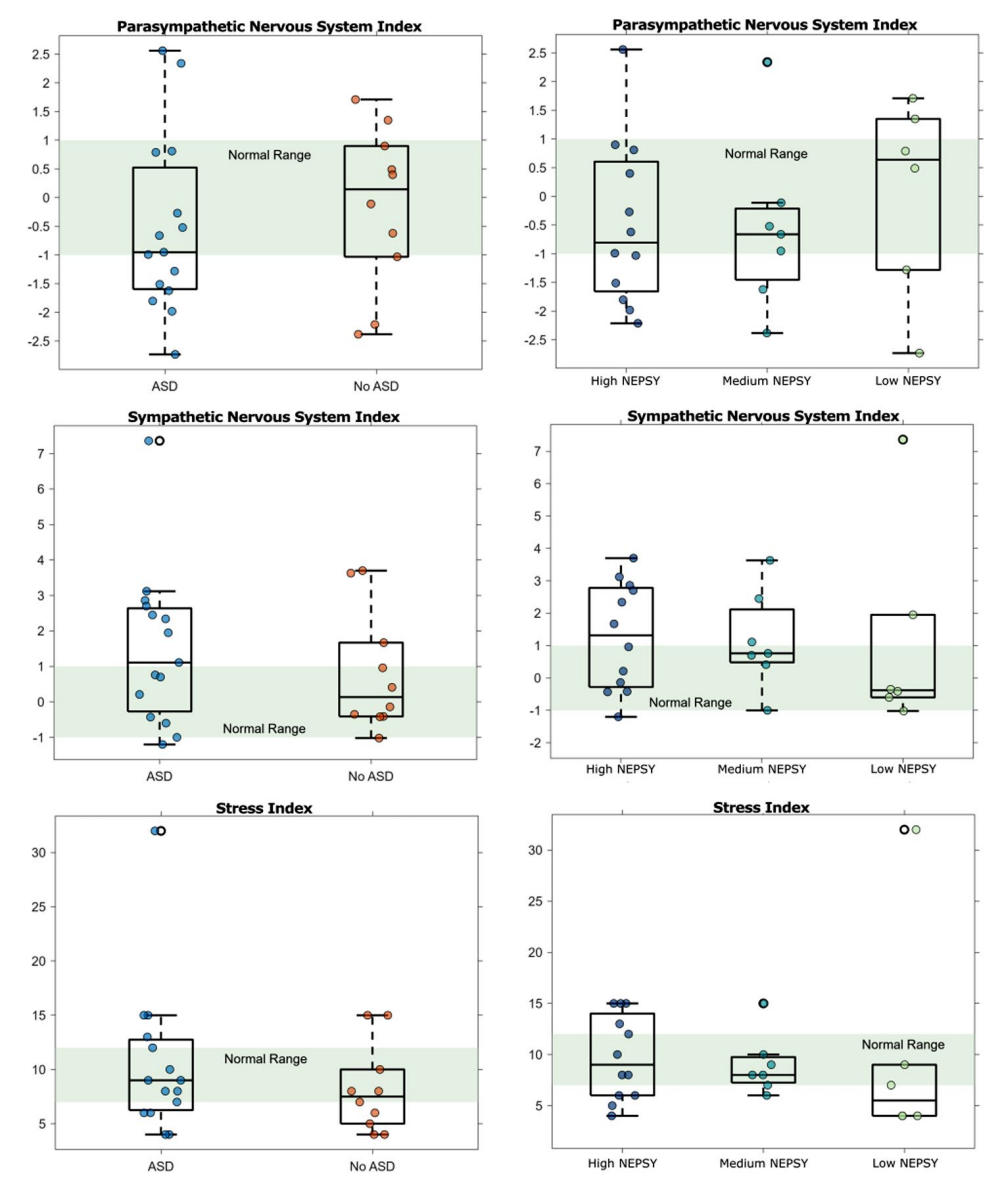
PNS, SNS, and Stress indices from Kubios across all driving tasks grouped by self-report of ASD or NEPSY ToM Score

We analyzed the given PNS, SNS, and stress indices from Kubios with the participants grouped by self-report of ASD and by NEPSY score. Figure 8 shows these indices across all of the driving stimuli. It is important to note that none of these indices are statistically significant, however, we observe trends that warrant further investigation.

Figure 8a shows the PNS index. A PNS index between 0 and 1 is in the normal range. A PNS index greater than 1 indicates quicker recovery from stressful stimuli to the “normal” state, whereas, a PNS index less than − 1 indicates slower recovery. As shown, the ASD group has a slightly lower median PNS index compared to the No ASD group. Additionally, we see that the “High NEPSY” group has a lower median PNS index and the “Low NEPSY” group has the highest median PNS index.

Next, Fig. 8b shows the SNS index. A SNS index between 0 and 1 is in the normal range. A SNS index greater than 1 indicates a quicker stress response to stimuli than average while an SNS index less than − 1 indicates a slower stress response. As shown, the ASD group has a higher median SNS index than the No ASD group. Additionally, the “High NEPSY” group also has a higher SNS index with a negative trend as the participant’s NEPSY score decreases.

Finally, Fig. 8c shows the Stress Index. A stress index between 7 and 12 is in the normal range. A stress index greater than 12 indicates higher than average stress whereas a stress index less than 7 indicates lower than average stress. We found a lower median stress index in the No ASD group than the ASD group. Additionally, we found lower levels of stress from the “Low NEPSY” group.

### Decisions

In the design of the driving tasks, there were two discrete possibilities: the participant will go before the other vehicle in the stimuli, or the participant will wait for the other vehicle to go first. However, there is not always a definitive “correct” answer. In this section, we will analyze the decisions the participants made in both the intersections and merging tasks based on both ASD grouping and NEPSY grouping.

**Fig. 9:**
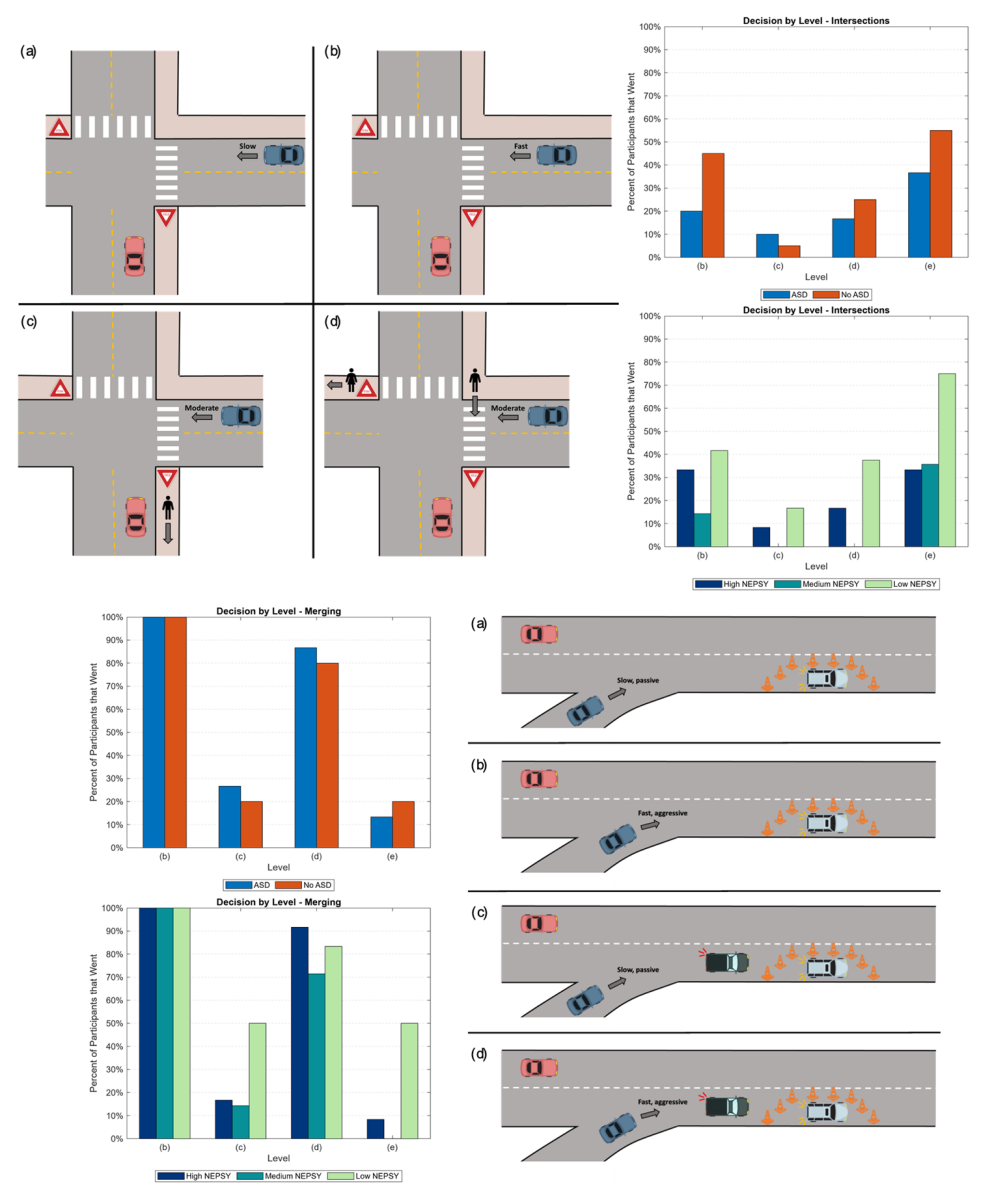
Decision the participants made by level based on grouping by self-report ASD and NEPSY ToM Score

Figure 9 demonstrates the decision by level for the intersection and merging tasks. In intersection task (b), the participant comes to an intersection with a yield, and a slow car is coming from the other direction. In this task, it would be safe for the participant to slow down, and then go before the blue car, however, it is not incorrect to wait. The bar graphs to the side show the percentage of participants that “went” before the blue car in these tasks. As shown, nearly double the number of participants in the No ASD group went compared to the ASD group in task (b). We also see that the “Low NEPSY” group went slightly more often than the “High NEPSY” group. In task (c), the blue car is now driving quicker. It is no longer safe for the participants to go, and they will likely get into a wreck if they do go. We see significantly fewer of the participants went in this task, however, the “Low NEPSY” group still goes slightly more than the other groups. In task (d), there is now a pedestrian that does not directly impact the task. We see the same trend we saw in task (b) in the ASD group goes more often than the No ASD group. However, this time we see a much larger jump in the amount of time the “Low NEPSY” group goes rather than waiting. Finally, in task (e), we introduce pedestrians crossing and blocking the blue cars’ path. We still see the same trend in the ASD and No ASD grouping. However, again, we see a big jump in the percentage of participants in the “Low NEPSY” group that go compared to the “High NEPSY” group. This indicates that the addition of pedestrians does not impact the decision of the “Low NEPSY” group as much as it impacts the group with the “High NEPSY” score.

In merging task (b), the participant comes upon an obstacle, and a slow-passive car needs to merge in front to avoid the obstacle. The participants can either slow down and let the blue car merge in front of them or go in front of the blue car. All of the participants went before the blue car in this level. In merging task (c), the blue car is now fast and aggressive. We see similar amounts of participants going before the blue car when splitting them based on self-reported ASD. However, when we split the participants by NEPSY score, we see that half of the participants in the “Low NEPSY” group go in this situation. In merging task (d), we introduce another vehicle that does not directly impact the task, and the blue car is still going slow. We see that the majority of participants go, and we do not see any notable differences between the groups. In the final merging task, (e), we increase the speed of the blue car, but we keep the additional vehicle that does not impact the task. We again see that half of the participants in the “Low NEPSY” group go compared to less than 10% of the participants in the “High NEPSY” group.

### Driving Theory of Mind Questionnaire

**Fig. 10:**
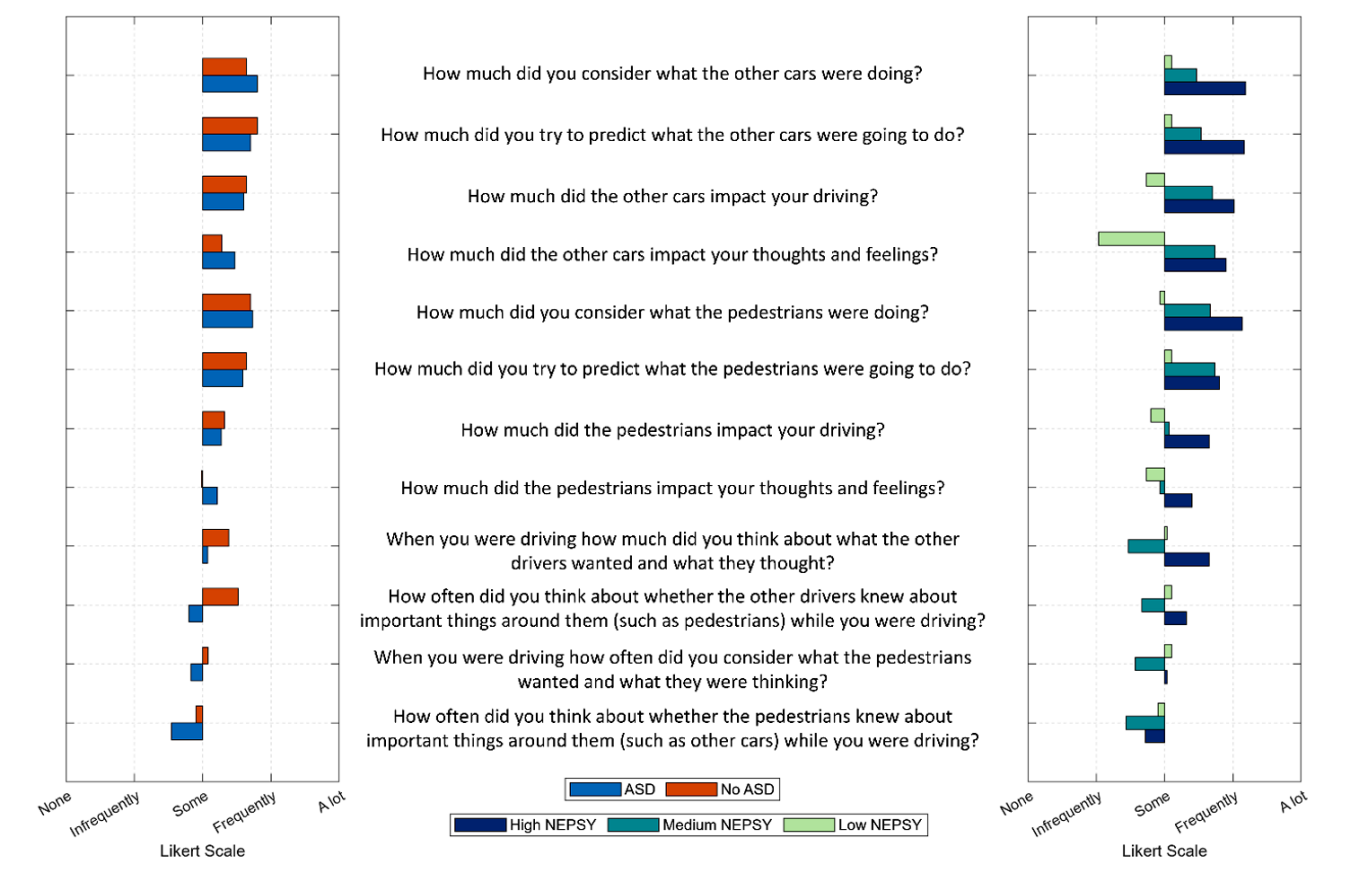
ToM Questionnaire administered after the driving tasks grouped by self-reported ASD and NEPSY ToM score

Following the completion of the driving tasks, the ToM questionnaire was administered. The questionnaire utilized a Likert scale ranging from − 10 to 10, where respondents indicated their level of agreement from ‘none’ to ‘a lot’. Figure 10 shows the responses to this questionnaire. The left plot shows the participant’s average responses grouped by self-reported ASD. We see negligible differences between the two groups. The right plot shows the participant’s average responses grouped by Nespy score. We see that the “High NEPSY” and “Medium NEPSY” groups consider pedestrians and other cars more than the “Low NEPSY” group.

## Discussion

In our study, we found comparable driving performance and attention between autistic and non-autistic novice drivers. However, recent statistics noted that only 24% of autistic adults are licensed compared to 75% of all adults. Our findings suggest that this discrepancy may not begin with initial driving skill, but conversely may be due to disparities in access to driving education and licensure pathways based on neurodevelopmental differences, highlighting broader societal implications for transportation equity and accessibility. Importantly, our study underscores the significance of Theory of Mind (ToM) abilities in driving performance. Notably, our study revealed more notable differences in driving performance, attention, stress responses, and decision-making among individuals with varying levels of Theory of Mind (ToM) abilities, as measured by NEPSY scores.

### Performance

At intersections, where drivers must navigate interactions with other road users, we observed significant differences in wheel rotation and speed among NEPSY score groups. Participants with lower NEPSY scores exhibited increased wheel rotation and slightly higher average speeds. We additionally found that all participants spent a similar amount of time braking and accelerating, however, the “Medium NEPSY” and “Low NEPSY” groups had more instances of hard accelerations and decelerations than the “High NEPSY” group which is indicative of unsmooth driving. These findings suggest that individuals with lower Theory of Mind (ToM) abilities may exhibit less smooth driving behaviors, manifested as overcompensation in steering, potentially leading to erratic or hesitant decision-making, which could adversely impact overall driving performance. Participants in the ASD group displayed similar driving patterns overall, indicating comparable performance in most aspects. However, they exhibited a higher frequency of hard accelerations, suggesting potential differences in driving style or response to environmental stimuli.

Similarly, in merging tasks, participants with lower NEPSY scores displayed higher median average speeds, reflecting potential difficulties in anticipating and adapting to traffic flow dynamics. While differences in wheel rotation were minimal, the observed increase in speed suggests a propensity for riskier driving behaviors among individuals with lower ToM abilities, possibly due to challenges in predicting the actions of other drivers and adjusting their behavior accordingly. Again participants with ASD exhibited a wider range of acceleration and braking times compared to neurotypical participants, suggesting variability in response to merging stimuli. Despite this variability, both groups exhibited a similar frequency of hard accelerations. Conversely, NEPSY score grouping reveals that participants with lower ToM abilities spend less time accelerating and braking overall. However, they compensate with more frequent hard acceleration and braking occurrences, indicating potential challenges in adapting to traffic flow dynamics.

### Attention

Our examination of attention revealed interesting trends, particularly regarding the focus on the road versus the speedometer. Participants with lower NEPSY scores demonstrated a reduced focus on the road and an increased fixation on the speedometer, indicating potential challenges in maintaining attention to critical environmental cues while driving. This discrepancy in attentional priorities could contribute to differences in driving performance in the NEPSY score groups.

### Physiology

While we found no significant differences in physiology regardless of how we grouped the participants, we found trends that warrant further investigation. The physiology results found that the ASD group had a lower PNS index, higher SNS index, and a higher stress index. This indicates that the ASD group was more stressed, got stressed quicker, and recovered slower than the No ASD group. We also found that the “Low Nepsy” group was less stressed, got stressed slower, and recovered quicker than the “High Nepsy” group. We hypothesize that this is because they are less concerned about the other vehicles and pedestrians in the stimuli due to having lower ToM. Additionally, in the prior section, we found that they focused on the speedometer more and the road less than the “High Nepsy” group which could indicate why they were not as stressed during the driving tasks.

### Decisions

Analysis of participants’ decisions at intersections and merging points revealed nuanced differences in risk-taking behavior based on NEPSY scores. Participants with lower NEPSY scores demonstrated a tendency towards riskier decisions, particularly in situations involving additional environmental complexities such as pedestrians and multiple vehicles. Whereas, the decisions between the ASD and No ASD group followed similar trends. These findings highlight the importance of ToM abilities in guiding adaptive decision-making and risk assessment during driving tasks.

### Driving Theory of Mind Questionnaire

Responses to the ToM questionnaire provided further validation of the relationship between NEPSY scores and perspective-taking abilities. Participants with higher NEPSY scores demonstrated a greater consideration of other road users’ intentions and perspectives, whereas the participants with lower NEPSY scores demonstrated lower consideration of pedestrians and other cars. This is reflected in the decisions they made and indicates why this group showed lower levels of stress.

Stress and anxiety while driving are commonly cited adversities to getting licensed in autistic drivers. This underscores the importance of addressing stress management techniques in driving education and training programs tailored for individuals with ASD. Moreover, our findings emphasize the necessity for driving education to encompass not only the mechanics of driving but also the social aspects of driving, including understanding and navigating complex social interactions on the road. Addressing these disparities in driving education and licensure pathways could contribute to greater inclusivity and accessibility in transportation systems, ensuring that individuals with autism spectrum disorder have equal opportunities to obtain driver’s licenses and participate fully in society.

Despite the insights gained from our study, several limitations should be considered. First, our study focused exclusively on novice drivers, and the driving scenarios were simulated rather than real-world experiences. While simulations allow for controlled experimentation, they may not fully capture the complexities of real-world driving situations. Additionally, future research could benefit from larger and more diverse samples, including experienced drivers. Furthermore, while NEPSY scores provided a measure of ToM abilities, they may not capture all aspects of social cognition relevant to driving performance. Other cognitive and behavioral factors, such as executive functioning and anxiety levels, were not directly assessed in our study but could influence driving behaviors. Despite these limitations, our study provides valuable insights into the relationship between ToM abilities and driving performance, highlighting the importance of considering social cognitive factors in driving education and licensure pathways, particularly for autistic individuals.

While our study sheds light on the relationship between Theory of Mind (ToM) abilities and driving performance, future research avenues could focus on addressing the challenges identified, particularly in stress management and the development of self-regulation driving systems that consider the social aspects of driving. By developing targeted interventions and innovative technologies, we can promote inclusivity, safety, and accessibility in transportation facilitating meaningful employment and fostering independence for everyone.

## Electronic supplementary material

Below is the link to the electronic supplementary material.

Supplementary Material 1

Supplementary Material 2

## Footnotes

1 The preference for identity-first language (autistic person) over person-first language (person with autism) in this context follows the principles advocated by many self-advocates and disability rights activists. However, it is important to recognize that language preferences may vary among individuals. (Taboas et al., 2023).
