# Supplementary figures and images for: Exploring the Intersection of Autism, Theory of Mind, and Driving Performance in Novice Drivers
Source: J Autism Dev Disord. 2024 Aug 28;55(6):2124–40. doi: 10.1007/s10803-024-06526-9 (PMC12069486; doi:10.1007/s10803-024-06526-9)

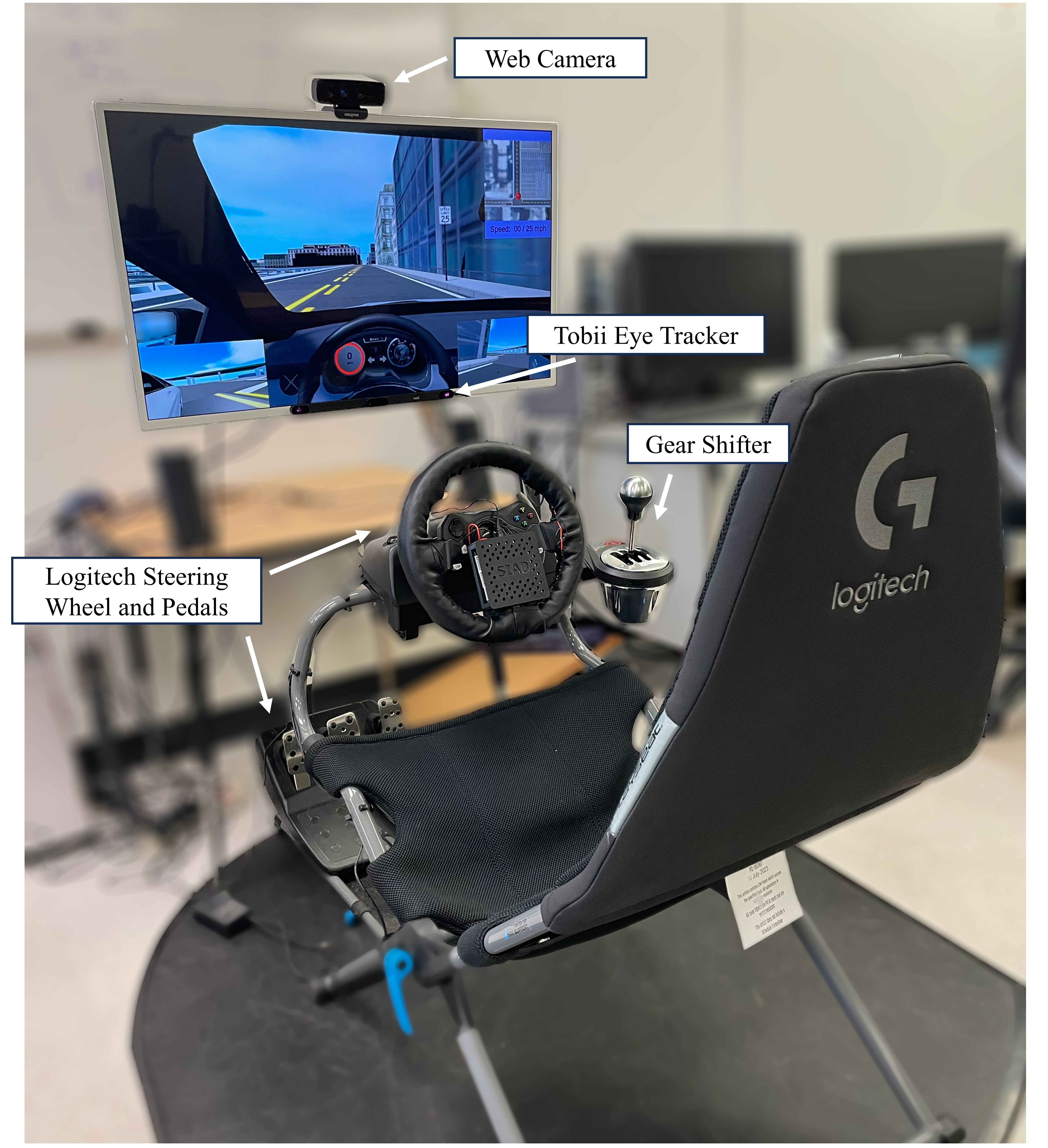

Supplement: Supplementary file 1 — Supplementary Material 1 [file 10803_2024_6526_MOESM1_ESM.png]

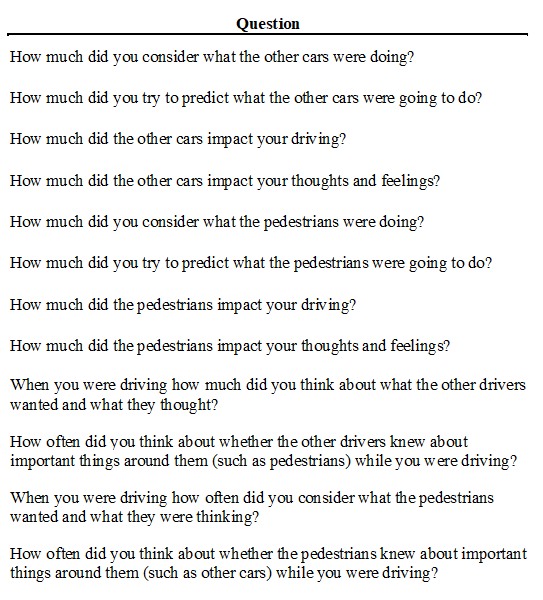

Supplement: Supplementary file 2 — Supplementary Material 2 [file 10803_2024_6526_MOESM2_ESM.png]
